# Thinking Outside the Black Box: Seven Guidelines for GenAI Use in the Ecological Sciences

**DOI:** 10.1002/ece3.74118

**Published:** 2026-08-11

**Authors:** Nina C. Ferrari, Emily E. Conklin, Amelia A. Fitch, Dustin G. Gannon, Rossana Macedo, Aidan Place, Hannah R. Sachs, Eugene Seo, Matthew J. Weldy, Matthew G. Betts

**Affiliations:** ^1^ Department of Forest Ecosystems and Society Oregon State University Corvallis Oregon USA; ^2^ National Audubon Society New York New York USA

**Keywords:** ecology, generative artificial intelligence, large language models, reproducibility, research ethics, scientific workflows

## Abstract

Generative artificial intelligence (GenAI) tools are increasingly incorporated into ecological workflows at every stage of the scientific process, from hypothesis generation and data collection to synthesis, analysis, manuscript preparation, and peer review. This rapid integration presents new opportunities for discovery but also poses fundamental challenges to the epistemological foundations of the field. We discuss how the uncritical adoption of GenAI risks eroding core principles of ecological inquiry, including idea generation, reproducibility, transparency, and engagement with natural systems. By prioritizing scale, speed, and statistical pattern detection over rigorous theory development and testing, GenAI may shift research away from theory‐driven questions and toward large‐scale data‐mining approaches. GenAI tools offer clear benefits when used thoughtfully; these include expanded access to information and the ability to synthesize across otherwise disparate disciplines and datasets. But overemphasis on these approaches could have adverse impacts on the field, including reducing reproducibility and ecological inference and reinforcing existing inequities in knowledge production. Here, we identify key challenges associated with GenAI integration in the field of ecology and provide seven actionable guidelines to support its responsible and effective use. We call for a concerted effort to engage in reflective and intentional use of GenAI in ecological research, resisting pressures to prioritize efficiency over the rigor, creativity, inclusivity, and societal relevance of our field.

## Graphical abstract

We identify key challenges associated with GenAI integration in the field of ecology and provide seven actionable guidelines to support its responsible and effective use. More broadly, we call for a concerted effort to engage in reflective and intentional use of GenAI in ecological research, resisting pressures to prioritize efficiency over the rigor, creativity, inclusivity, and societal relevance of our field.

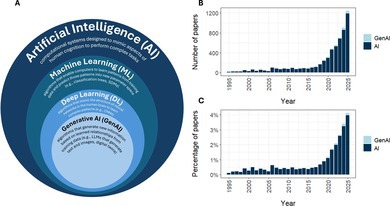

## 1 Introduction

While the term “artificial intelligence” (AI) is now common vernacular, ecologists have been using approaches that are considered under the umbrella of AI for decades, even if they were not described as such. AI methods have been used to classify patterns (Breiman 2001), predict ecological processes, and analyze complex datasets (Christin et al. 2019; Olden et al. 2008; Recknagel 2001), with applications ranging from evolutionary modeling to image and acoustic data processing. However, recent advances have led to the rapid development and proliferation of a new branch of AI referred to as Generative AI (GenAI). This class of methods marks a transition toward models that can perform complex tasks across multiple domains through common language interfaces, opening the door to their use across a wide range of ecological research applications. Broad examples of GenAI include large language model (LLM)‐driven chatbots and agentic systems that can execute tasks at the directive of an LLM. Applications developed specifically for ecological use include programs that simulate images of landscapes or organisms (Frazier and Song 2024; Rafiq et al. 2025; Richards et al. 2024) and virtual ecosystem replicas (“digital twins”) (De Koning et al. 2023; Tan and Cheng 2024).

GenAI has already led to paradigmatic shifts across disciplines and can facilitate many steps of the scientific process, including developing ideas, collating information, generating code for analysis, and writing and editing manuscripts. This capability has been touted as having potential to push the field forward by increasing scientific discovery rates (Agathokleous et al. 2023; Bianchini et al. 2022; Doi et al. 2023; Frazier and Song 2024; Han et al. 2023; Li and Guo 2025; Smith et al. 2024; Wang et al. 2023; Wilson 2024). However, overuse of these tools poses fundamental challenges to the epistemological foundations of research. The rapid proliferation of GenAI tools, combined with substantial media attention surrounding their development (Zwingmann 2025) and institutional and federal funding incentives (Mervis 2026), has accelerated the adoption of GenAI tools across disciplines. A survey conducted by Elsevier in 2025 found that 58% of researchers used AI tools for work, up from 37% in 2024 (Elsevier 2025). GenAI is already being widely used in the ecological sciences for coding, analysis, and text editing and has potential for expanded use in other steps in the scientific process (Doi et al. 2023; Frazier and Song 2024; Rafiq et al. 2025). As an extreme example, pipelines have been developed that fully automate scientific research from conception to publication (Lu et al. 2026).

While GenAI applications can be powerful, their outputs are sensitive to the quality and composition of the data on which they are trained. The companies that develop these technologies use massive amounts of data scraped from the internet to train models prior to public release (Kahlon and Singh 2026; Liang et al. 2026). Therefore, the content of the training data, and the relationships the models learn from these data, are fundamentally opaque to users (Brown et al. 2020; Han and Zhang 2025; Naveed et al. 2024), making it difficult to know under what circumstances outputs may be biased or misleading (Boyko et al. 2023; Gougherty and Clipp 2024; Liesenfeld et al. 2023; Reyhani Haghighi et al. 2023). Furthermore, stochastic variation in outputs from GenAI, where the same prompt or query can yield different responses, raises concerns about transparent and reproducible workflows (Barrie et al. 2025; Bisbee et al. 2024; Liesenfeld et al. 2023; Srinath and Ram 2026). Unless users apply appropriate thought and caution, the opacity and obscurity of outputs could have negative downstream consequences on reproducibility and bias when GenAI is used to guide ecological research.

**FIGURE 1:**
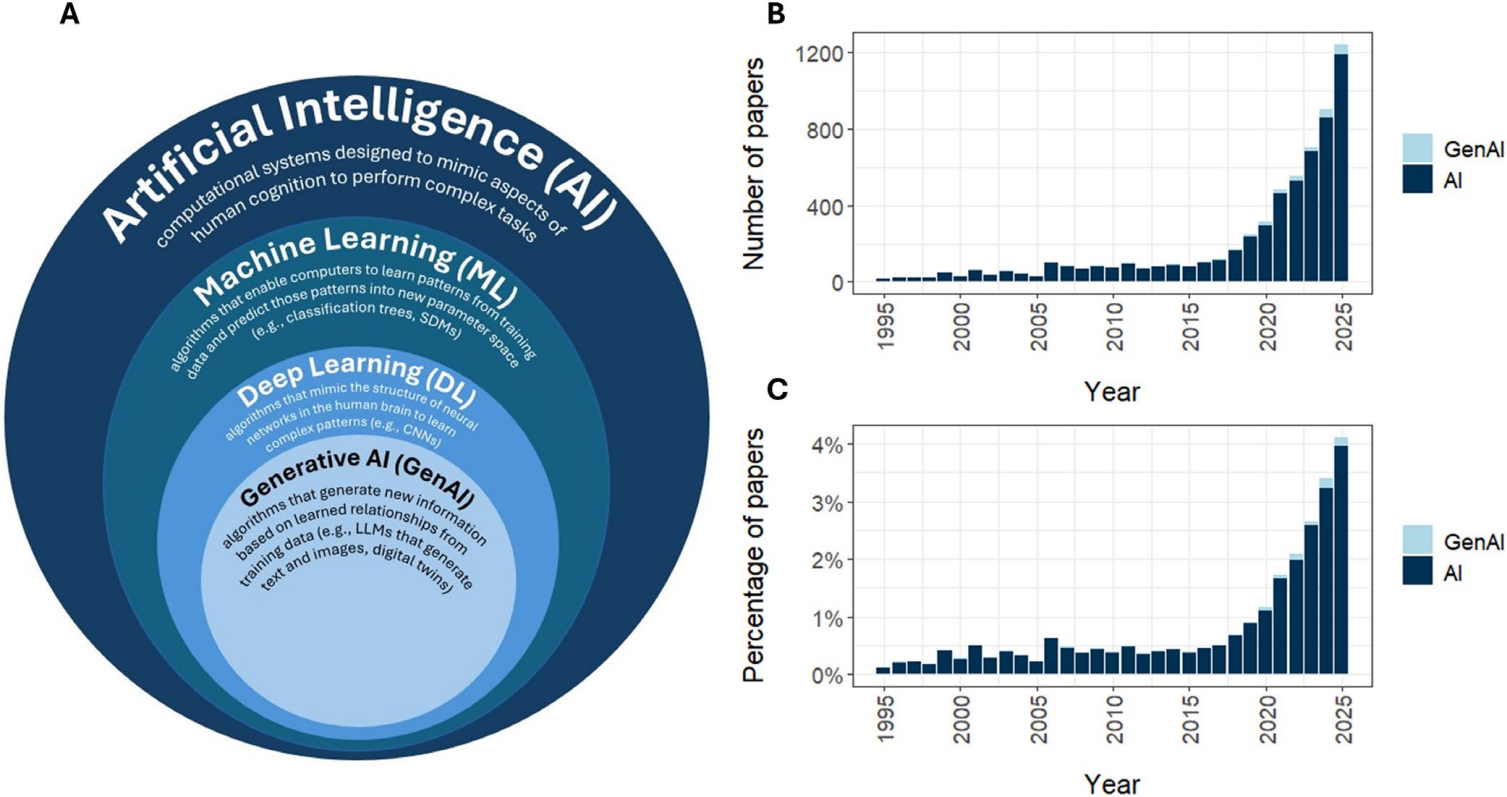
(A) Nested AI subfields (adapted from Frazier and Song 2024). (B) Absolute count of AI‐related ecology publications per year and (C) percentage of AI‐related ecology publications per year, expressed as stacked bars separating non‐generative AI (dark blue) and generative AI (light blue) papers. The total number of ecology papers per year was calculated from Web of Science subject categories [search term: (WC=(Ecology))]. Non‐generative AI papers were identified using the search term (WC=(Ecology)) AND (TS=(AI) OR TS=(artificial intelligence) OR TS=(machine learning) OR TS=(deep learning) OR TS=(neural network)) NOT (TS=(generative AI) OR TS=(GenAI) OR TS=(large language model) OR TS=(LLM)). Generative AI papers were identified using the search term (WC=(Ecology)) AND (TS=(generative AI) OR TS=(GenAI) OR TS=(large language model) OR TS=(LLM)). Literature searches were conducted on March 6, 2026.

With the widespread adoption of GenAI technologies in ecological research (Figure 1A), the number of AI‐related papers in ecology has increased rapidly over the past 5 years, even after accounting for general increases in papers over time (Figure 1B,C). Given the power and potential for unintended bias, this widespread adoption warrants a moment of pause to consider how researchers engage with GenAI. However, there has been little guidance provided thus far on best practices for GenAI use in the field of ecology (Messeri and Crockett 2024; Mishra 2026). Specifically, what practices are necessary for GenAI use while maintaining research integrity? Can GenAI be used in fair and equitable ways? How can we maintain reproducible workflows when using inherently stochastic generative processes? If left unaddressed, we caution that these new tools have the potential to undermine the foundations of the scientific process. Here, we provide an overview of some potential challenges with respect to GenAI use in ecology, as well as the scientific community more broadly, and offer actionable guidelines for how ecologists can engage with these technologies while safeguarding scientific integrity.

### 1.1 Lead With the Foundations of Ecological Inquiry

The scientific method begins with observing patterns in natural systems, generating questions about those patterns, and posing hypotheses about the processes driving those patterns (Betts et al. 2021; Chamberlin 1965; Wolff and Krebs 2008; Zilsel 1941). In ecology, this process is rooted in natural history, from Linnaeus' taxonomic documentation to Darwin's observations that shaped evolutionary theory (Darwin 1888; Linnaeus 2024). Developing a deep understanding of ecological theory and the natural history of a study system requires extended time in the field (Anderson 2017; Jones 2026; Laughlin 2024; Stengers 2018), a luxury not often afforded to researchers in today's fast‐paced academic environment (Edwards and Roy 2017; Lane 2017; Tewksbury et al. 2014). GenAI has been proposed as a tool for automating steps of the scientific process from developing research questions to generating hypotheses (Lu et al. 2026). Some proponents argue that GenAI will “optimize” the scientific process and lead to acceleration of scientific discovery rates by using LLMs to pose novel research questions and propose new theories (Cohrs et al. 2025; Huang et al. 2025; Singh 2015; Xiong et al. 2024). While this technology may increase the rate of knowledge generation, there are downsides to consider (Alai 2004; Chubb et al. 2022). Reliance on GenAI for research question and hypothesis generation may contribute to a growing disconnect from natural history field observations, a phenomenon previously referred to as the “natural history gap” and an “extinction of experience” (Irwin 2026; Kusumegi et al. 2025; Labonté and Trueman 2025; Soga and Gaston 2025). Given the opacity of the GenAI training process, ideas generated by LLMs may not necessarily be rooted in a strong ecological rationale (Dorm et al. 2026). More critically, we may limit our ability to gut‐check or ground‐truth those outputs without time in the field to observe (Han et al. 2023).

Furthermore, idea generation in science rarely occurs in isolation. Productive brainstorming is a collaborative process aided by mentors and colleagues who challenge thought and push ideas forward (Ellemers 2021; Sonnenwald 2007). The agreeable tone of many LLMs is antithetical to this process (Cheng et al. 2026); instead of proposing alternative perspectives, they can corral conversation into capitulatory consensus. We argue that novel ecological questions are most likely to emerge through direct observation of nature and engagement with human colleagues who will challenge our ideas, share experience, and advance our thinking. While GenAI offers the potential to aid in various tasks throughout the scientific process, as a field, we should ensure that we prioritize using these tools to free up time for, rather than outsource, careful observation, idea generation, and reflection. Overreliance on GenAI for these fundamental steps of the scientific process could shift the field away from place‐based observations and hypothetico‐deductive reasoning, which are foundational to scientific rigor and integrity.

We recommend that ecologists engage in periods of observation and reflection in their study systems. Avoid relying on LLMs to inform and generate research questions and hypotheses; instead, rely on ecological theory and in situ observations. Make time to return to field‐based observations and synthesize these perspectives to generate research questions grounded in one's study system.

### 1.2 Understand Appropriate Use Cases

The development of novel methods can result in a flurry of adoption, often without sufficient understanding of their limitations or applicability to particular use cases (Blanchet et al. 2020; Steel et al. 2013). While GenAI is already emerging as a tool in ecologists' toolboxes, its unique intersection of user accessibility and black‐box complexity makes it a potential candidate for misapplication and misinterpretation (Messeri and Crockett 2024; Zwingmann 2025). Given the probabilistic nature of its outputs (Pournemat et al. 2025), limited replicability (Bisbee et al. 2024), and environmental impacts (Li et al. 2025), careful consideration should be taken when deciding if GenAI is a good fit for an ecological application. The distinction between appropriate and inappropriate uses of GenAI in ecology will not be binary, but rather a gradient spanning from simple to complex applications; discernment will depend on the specific user and task at hand.

As GenAI becomes further integrated into the field, ecologists should avoid the justification of its use purely based on novelty and perceived complexity (i.e., “AI machismo”) (McGill 2012; Norman et al. 2026), especially if sufficient and simpler tools already exist. For example, a study from Berger‐Tal et al. (2024) compared ChatGPT3.5 to a non‐generative ML algorithm in screening papers for a literature review and found them to have comparable performance. In situations where GenAI approaches do not provide any particular heuristic or analytical advantage, scientists should consider opting for simpler, more interpretable, replicable, and less environmentally costly alternatives. We recommend that researchers think critically about whether GenAI tools are appropriate for their particular use case, develop a strong understanding of these methods and tools to avoid misapplication, and carefully weigh the risks and benefits. When non‐GenAI alternatives are available and sufficient, we encourage opting for them instead.

### 1.3 The Fundamentals of Statistical Inference Still Apply

One of the many intriguing use cases for GenAI is harmonizing small publicly available datasets from across similar but discrete studies. For example, iChatBio (Elliott et al. 2025) is an LLM that compiles biodiversity data from known sources like Global Biodiversity Information Facility (GBIF) and iNaturalist based on plain‐language prompts from the user. Indeed, GenAI tools can make it easier to assemble, wrangle, and analyze data that have been collected using different naming conventions, units, and data storage formats (Elliott et al. 2025). While this capability poses tremendous opportunity to synthesize the current state of knowledge on a given topic, it does not negate the need to understand the provenance and study design of each data source.

Ecological inference has long relied on carefully planned sampling designs and suitable analyses intended to address predetermined hypotheses (Albert et al. 2010; Betts et al. 2021; Williams and Brown 2019). Ecologically informed study design allows for the collection of appropriate data to answer specific research questions, resulting in reliable and interpretable estimates of parameters and uncertainty (Popovic et al. 2024; Williams and Brown 2019). Disregarding underlying data structures can lead to biased inferences and misleading results, regardless of sample size (Christie et al. 2019; Foster et al. 2021). Unfortunately, though discovery and retrieval can be made easier by these tools, many online data repositories may lack the documentation needed to understand sampling constraints, making information on the original study design difficult or impossible to recover via automated retrieval (Liao and Wortman Vaughan 2024). It is important therefore to verify that multiple datasets can be combined in a meaningful way. Human discretion remains critical to assembling data for downstream analysis.

Recent advances in GenAI have also enabled the use of augmentation to bolster limited ecological datasets (Capua and De Cristóforis 2024; Crocker et al. 2024). While approaches such as creating augmented datasets may increase sample size and apparent coverage, they do not inherently resolve issues of representativeness. Augmented data are ultimately constrained by the structure and biases of the original data and may therefore reproduce or even amplify existing patterns rather than improve generalization (Frazier and Song 2024; Rillig et al. 2024; Shorten and Khoshgoftaar 2019; Van Den Goorbergh et al. 2022). Careful evaluation is therefore required to ensure that augmentation does not introduce misleading or ecologically unrealistic variation.

GenAI, and particularly agentic systems, can also be used to execute tasks and call different applications to conduct data analysis. We see merit in the idea of democratizing data analysis skills with the use of natural language to interface with computational engines (Gadde 2024). However, researchers should prioritize developing a comprehensive understanding of the system, data, and analytical methods being applied, even if processing and computation can be directed and completed by a GenAI agent. To do otherwise risks shifting research away from theory‐grounded hypotheses and toward a “test everything” data‐mining approach. For example, mining large biodiversity databases with GenAI tools could potentially generate novel hypotheses about species interactions that would be otherwise difficult to identify, but those hypotheses may instead reflect spurious patterns detectable in large datasets rather than meaningful biological relationships, essentially “p‐hacking” at computer speed (Twining and Kellner 2025).

Lastly, the sheer volume of relationships and tests that can be explored in short time frames with GenAI is a potential cause for concern. Historically, the time required to collect or at least assemble and analyze large datasets provided a *de facto* check on investing in half‐baked ideas, where the friction of the process itself discouraged the pursuit of underdeveloped research directions (Chen and Schmidt 2024). With the efficiencies of GenAI reducing that friction, a single research group could run successive analyses with little time investment. This low cost of iteration, combined with high pressure on scientists to publish impactful results, raises concerns that a higher proportion of spurious findings will inundate our fields (Chen et al. 2024). Allowing time and space for deep thought, justification, and documentation of downstream analytical decisions will make efforts more transparent, reproducible, and rigorous (Powers and Hampton 2019).

The most effective use of GenAI for data analysis in ecology will come from pairing these emerging tools with the careful statistical considerations that underpin reliable ecological inference. We recommend that, when using GenAI‐compiled data, researchers collaborate with the original data collectors and consult accompanying metadata to ensure that study design assumptions and nuances of the data are appropriately incorporated into analyses. In cases where the underlying sampling design is unknown, data may be better suited for exploratory analysis that then is rigorously tested with newly collected data to enable credible ecological inference. In instances where GenAI is used to generate augmented datasets, ensure augmentations are representative of the range of conditions expected. We recommend that researchers think deeply about their analysis plan prior to considering the use of GenAI tools.

### 1.4 Consider Who Benefits From Increased Accessibility

Open access to scientific data and code benefits science and society by allowing others to reanalyze and integrate the data, fostering the democratization of knowledge. Combining accessible datasets can lead to new insights and answer novel questions (Mills et al. 2015). However, there is a long‐recognized tension between the benefits of open data and their potential misuse, especially with respect to Indigenous data sovereignty, rare species locations, and other potentially sensitive, but not legally or editorially protected, datasets (Bowser et al. 2017; Jones et al. 2025; Lindenmayer and Scheele 2017; McCartney et al. 2022). The recognition that “open” is not synonymous with “ethical” has led to the formation of data use principles such as Collective Benefit, Authority to Control, Responsibility, and Ethics (CARE; Carroll et al. 2020; Marriott et al. 2025). With the potential of GenAI to analyze data at massive scales, there are concerns about the amplification of unethical data misuse (Pasquetto et al. 2024). Additionally, following Smith et al. (2024), we caution against sharing with LLMs any code, data, or writing that may contain sensitive or confidential data.

Beyond data access issues, it is also worth monitoring the privatization of aspects of the scientific workflow, including methodologies, models, and training processes. For instance, Google recently introduced noncommercial quota tiers for Google Earth Engine projects (Google 2026). These tiers allocate monthly computational resources at different levels, with higher‐use options requiring additional conditions. There is a growing ecosystem of other AI‐supported subscription products built around knowledge‐based workflows for reviewing literature (LeapSpace), writing (Grammarly, Jenni AI), research (Connected Papers, Research Rabbit), analysis (Claude Code, Antigravity), and visualization (Figma, Canva). It is worth considering the ramifications on transparency, reproducibility, and intellectual control if any of these tools become “load‐bearing” in the production of scientific knowledge, especially as costs could inhibit reproducibility. Although this dilemma is certainly not new ground nor unique to GenAI software (Ince et al. 2012), we suggest that given the potential widespread proliferation of GenAI tools at multiple steps of the scientific process, it is well worth revisiting the issue.

We recommend that ecologists stay informed of current standards of data stewardship, governance, and use as they engage with GenAI tools, and practice due diligence in assessing the provenance and any ethical use considerations of GenAI‐provided data. We recommend authors transparently report the use of LLMs in any steps of the research process including brainstorming (conversations with chatbots), data download, data analysis (model cards), and writing in the supporting material to the paper. We also encourage the scientific community to develop a formal code of ethics for engaging with GenAI‐collated data.

### 1.5 Strive for Accurate and Inclusive Writing

GenAI tools have the potential to amplify existing issues in misattribution of intellectual property, plagiarism, and bias. These tools can be helpful in early steps of gathering information, and when used thoughtfully can increase the reach of literature searches by collating “grey literature” and unpublished reports that may otherwise be neglected (Berger‐Tal et al. 2024; Mammides et al. 2026; Smith et al. 2024). However, the tendency for hallucination and proliferation of inaccuracies in GenAI is well‐documented (Ji et al. 2023; Kalai et al. 2025; Martino et al. 2023). In terms of intellectual ownership and credit, overreliance on LLM outputs can result in citation errors (Byun et al. 2024; Naddaf and Quill 2026), and although LLMs show potential for generation of novel ideas (e.g., Thaler 2026), any assertion of novelty should be backed up by careful expert review to avoid potential plagiarism (Gupta and Pruthi 2025).

It is plausible that citation errors will not be as much of a concern in the future, as methods for mitigating hallucination continue to improve (Farquhar et al. 2024). However, there are concerns of bias amplification even when LLMs provide real and relevant citations. Publication rates in the field of ecology are biased by geographic area, biome, human settlement, country wealth (Hughes et al. 2021; Martin et al. 2012), gender (Fontanarrosa et al. 2024), language (Konno et al. 2020), and race and ethnicity (O'Brien et al. 2020). These disparities are further shaped by financial barriers to publication and access, including the high costs of open‐access publishing and subscription‐based journal paywalls (Ayeni and Larivière 2025; Fontúrbel and Vizentin‐Bugoni 2021). Because GenAI systems are trained on accessible digital text, they may disproportionately draw from open‐access literature that is more readily available, while underrepresenting work published in less accessible venues or by under‐resourced researchers. LLMs are inadvertently trained on these biased datasets, and so rather than acting as a neutral or objective third party, these tools tend to perpetuate the power structures that built them (Ho 2023), show heightened citation bias as compared to human researchers (Algaba et al. 2025), and reproduce institutional bias patterns in peer review (Howell et al. 2025). For example, Urzedo et al. (2024) found that when asked questions about conservation, two‐thirds of ChatGPT's answers drew on the expertise of male researchers at United States universities, while largely ignoring the expertise of Indigenous and community knowledge holders and researchers from low‐ and middle‐income countries. The unreflective use of GenAI tools in the writing and citation process may perpetuate existing representation biases, potentially further excluding underrepresented groups. Additionally, the characteristically sycophantic tone of LLM outputs risks reinforcing existing biases rather than challenging assumptions through fact‐based dialog (Cheng et al. 2026). A shift toward amplifying the “voice” of tools such as LLMs in ecology without careful consideration of implicit bias risks an averaging or narrowing of represented viewpoints, values, and backgrounds.

We recommend that authors follow existing best practices for mitigating citation biases. Authors should not use LLMs as their sole source of locating and compiling citation information; LLMs are not a replacement for sound citation management and practices; any citation suggestions provided by AI tools should be carefully vetted and considered. Recognize the inherent biases of AI, and carefully consider biases in representation, viewpoint, background, and opinion of generated ideas and citation lists.

### 1.6 Emphasize Critical Thinking Over Efficiency

Academia, like many sectors of society, has increasingly shifted toward a productivity‐driven framework. The “publish or perish” culture, combined with competitive grant funding and tenure systems that heavily reward publication metrics, has intensified pressure on researchers to produce high‐impact work quickly (Edwards and Roy 2017; Lortie et al. 2012). As a result, efficiency and output often take precedence over intentional “slow science” (Fischer et al. 2012; Stengers 2018). GenAI has emerged within this productivity‐oriented environment as a tool that can accelerate nearly every stage of the research process from idea generation, literature synthesis, data curation, analysis, and manuscript drafting. However, overreliance on GenAI can reduce critical thinking and innovation (Choudhuri et al. 2026; Duhaylungsod and Chavez 2023; Jafari and Vassileva 2026; Lee et al. 2025; Zhai et al. 2024). For example, in a recent study, participants assigned an essay‐writing task assisted by an LLM showed weaker neural connectivity relative to writers who did not use an LLM (Kosmyna et al. 2025). Furthermore, users who rely heavily on GenAI for certain tasks like coding and writing may be overlooking the benefit of creatively working through problems. Outsourcing these tasks with GenAI has a time and a place, but overreliance may reduce our ability to think through certain problems and ruminate on ideas. The computer scientist Lamport (2014) summed it up nicely when he said:

*“If you're thinking without writing, you only think you are thinking.”*

The convenience that LLMs offer can also shift perception on when and how to engage with information (Duhaylungsod and Chavez 2023). For instance, the output of LLMs is better understood as a recombination of patterns from training data (i.e., “stochastic parroting”) than as the generation of factual information to learn from (Bender et al. 2021; Ji et al. 2023; Kalai et al. 2025). Furthermore, LLMs may inadvertently constrain novel ways of thinking. For example, a researcher could read a single influential paper that fundamentally changes the way they think about a subject, but if they were to read an LLM's response about the same topic, it would likely present a consensus view of the average or most probable information based on the historical subject matter of its training data. Overreliance on these consensus outputs could stagnate scientific thinking (Van Quaquebeke et al. 2025). Further, while LLMs may be able to pose novel ideas about a particular subject matter, they cannot necessarily discern which are interesting and worth pursuing. When adopted hastily in pursuit of research output over understanding, GenAI tools risk diluting the intellectual rigor that gives scientific knowledge its credibility.

As tech companies continue to advance GenAI technologies with goals of artificial general intelligence and superintelligence, touted to surpass human cognitive capabilities (Buttazzo 2023; Gulchenko 2024), we expect that machine thinking will increasingly diverge from human thinking, making deep, human reasoning, particularly when derived from in situ natural history observations, ever more valuable. As ecologists are called to address more pressing and complex problems, reliance on GenAI to find novel and creative solutions to ecological problems may be limited (Alai 2004; Cheng et al. 2026). While novelty can emerge via synthesis within the bounds of what is already known about systems (i.e., integrating previously disparate subfields within or outside of ecology), some new discoveries likely lie well beyond what exists in the current universe of ecological and scientific understanding. Thinking outside of the “black box” may be necessary to tackle the challenging ecological problems ahead.

We recommend that ecologists consider when and how they engage with LLMs and encourage critical thinking, skill development, problem solving, and learning alongside productivity. We encourage pondering ideas and grappling with problems on your own or with collaborators prior to interacting with GenAI chatbots.

### 1.7 Consider the Role of GenAI Beyond Research

Most ecologists occupy roles beyond research (administrators, journal reviewers and editors, educators, mentors, community liaisons, and more). Therefore, it is imperative that researchers take time to familiarize themselves with the uses and potential abuses of GenAI tools across other aspects of their work. Decisions about whether and how to integrate GenAI extend beyond individual workflows and have implications for institutional norms, professional standards, and the training of future scientists.

The role of GenAI in education and mentorship remains a subject of debate, research, and rapid policy‐setting (Milano et al. 2023; Peláez‐Sánchez et al. 2024). While these tools can support learning by providing accessible explanations, coding assistance, and writing feedback (Yigci et al. 2025), overreliance may decrease engagement (Choi et al. 2023) and hinder the development of foundational critical thinking and problem‐solving skills (Duhaylungsod and Chavez 2023). Mentors and instructors must therefore carefully consider when GenAI use enhances learning versus when it replaces learning, and should establish clear expectations for learners that prioritize skill development and conceptual understanding. In particular, mentors should aid mentees in developing basic research skills before integrating GenAI into workflows.

In the context of editorial processes, GenAI tools may impact the scope, volume, and quality of submitted manuscripts, as well as the process of peer review and publishing. We expect that in the near future there will be an influx of GenAI‐assisted ecology syntheses. This paradigm has the potential to extend the inference space of ecology as a whole, but the use of AI to generate manuscripts, reviews, or editorial decisions may undermine trust in the scientific process if not disclosed and critically evaluated (Öztürk et al. 2025; Van Quaquebeke et al. 2025). Reviewers and editors should consider whether AI‐assisted outputs meet the standards of rigor, originality, and transparency expected in scientific publishing. Additionally, editors and reviewers should consider the importance and impact of primary research compared to GenAI syntheses. Following Hosseini and Horbach (2023), we encourage authors, reviewers, and editors to disclose whether and how LLMs were used and accept full responsibility for issues relating to data security, plagiarism, and accuracy. Journal editors should consider mandating these disclosures, and indeed many journals already have GenAI policies in place for both authors and reviewers. With the rapid development and proliferation of GenAI tools, it may be necessary to regularly review and update these policies.

Administrative and institutional decisions regarding GenAI, including procurement of tools and expectations for use, will shape how these technologies are adopted across the field. Increasing reliance on proprietary platforms raises concerns about transparency, reproducibility, and the privatization of scientific infrastructure. Administrators should carefully consider use cases and trade‐offs before using, recommending, or requiring GenAI tools in their organizations. We recommend that ecologists proactively engage their universities, research institutions, professional societies, departments, and lab groups to draft policies around the use of GenAI, with specific language to protect researchers who actively choose the “slow science” approach and opt out of automating certain processes.

Finally, ecologists often serve as intermediaries between science and society. On the one hand, GenAI could aid in the “science communication training gap” by helping scientists adapt their academic writing to broader audiences (Barel‐Ben David et al. 2026). On the other hand, the use of GenAI in communication, outreach, and decision‐making carries risks of misinformation, oversimplification, or misplaced authority if AI‐generated outputs are treated as expert knowledge (Schäfer et al. 2024). Improving and maintaining public trust requires clear communication about the role of AI in scientific work and continued emphasis on human expertise, responsibility, and judgment.

We recommend that ecologists think broadly about the integration of GenAI across their professional roles and proactively engage in developing norms, policies, and ethical guidelines that extend beyond research alone. Researchers should consider the role of GenAI tools in administration, mentorship, education, peer review, and public outreach and communication.

**Guidelines for GenAI Use in Ecology**

1. Lead with the foundations of ecological inquiry: We recommend that ecologists engage in periods of observation and reflection in their study systems. Avoid relying on LLMs to inform and generate research questions and hypotheses; instead, rely on ecological theory and in situ observations. Make time to return to field‐based observations and synthesize these perspectives to generate research questions grounded in one's study system.
2. Understand appropriate use cases: We recommend that researchers think critically about whether GenAI tools are appropriate for their particular use case, develop a strong understanding of these methods and tools to avoid misapplication, and carefully weigh the risks and benefits. When non‐GenAI alternatives are available and sufficient, we encourage opting for them instead.
3. The fundamentals of statistical inference still apply: We recommend that, when using GenAI‐compiled data, researchers collaborate with the original data collectors and consult accompanying metadata to ensure that study design assumptions and nuances of the data are appropriately incorporated into analyses. In cases where the underlying sampling design is unknown, data may be better suited for exploratory analysis that then is rigorously tested with newly collected data to enable credible ecological inference. In instances where GenAI is used to generate augmented datasets, ensure augmentations are representative of the range of conditions expected. We recommend that researchers think deeply about their analysis plan prior to considering the use of GenAI tools.
4. Consider who benefits from increased accessibility: We recommend that ecologists stay informed of current standards of data stewardship, governance, and use as they engage with GenAI tools, and practice due diligence in assessing the provenance and any ethical use considerations of GenAI‐provided data. We recommend authors transparently report the use of LLMs in any steps of the research process including brainstorming (conversations with chatbots), data download, data analysis (model cards), and writing in the supporting material to the paper. We also encourage the scientific community to develop a formal code of ethics for engaging with GenAI‐collated data.
5. Strive for accurate and inclusive writing: We recommend that authors follow existing best practices for mitigating citation biases. Authors should not use LLMs as their sole source of locating and compiling citation information; LLMs are not a replacement for sound citation management and practices; any citation suggestions provided by AI tools should be carefully vetted and considered. Recognize the inherent biases of AI, and carefully consider biases in representation, viewpoint, background, and opinion of generated ideas and citation lists.
6. Emphasize critical thinking over efficiency: We recommend that ecologists consider when and how they engage with LLMs and encourage critical thinking, skill development, problem solving, and learning alongside productivity. We encourage pondering ideas and grappling with problems on your own or with collaborators prior to interacting with GenAI chatbots.
7. Consider the role of GenAI beyond research: We recommend that ecologists think broadly about the integration of GenAI across their professional roles and proactively engage in developing norms, policies, and ethical guidelines that extend beyond research alone. Researchers should consider the role of GenAI tools in administration, mentorship, education, peer review, and public outreach and communication.

## 2 Conclusion

GenAI is arriving as ecology is already grappling with accelerating environmental change, an ever‐increasing “fire hose” of available data, and growing pressure to produce actionable science at speed. In this context, GenAI offers powerful new tools with the potential to enhance efficiency, expand the scope of synthesis, and enable researchers to ask questions at spatial and temporal scales that were previously inaccessible (Doi et al. 2023; Frazier and Song 2024). Early applications suggest that GenAI can support the integration and synthesis of heterogeneous ecological data, assist in generating hypotheses, and accelerate the development of models and analytical workflows (Li and Guo 2025; Smith et al. 2024). These capabilities are particularly relevant for ecology, where fragmented datasets, scale limitations, and complex systems often constrain inference. In addition, GenAI has the potential to enhance communication and accessibility by translating complex ecological information across formats and audiences, supporting both interdisciplinary collaboration and engagement beyond academia (Wilson 2024). While many of these applications remain nascent, they build on a broader foundation of GenAI‐driven advances in ecological inference (Agathokleous et al. 2023; Bianchini et al. 2022). When used thoughtfully, GenAI offers promising methods to extend the scope and reach of ecological research in a rapidly changing world.

However, as we have outlined, these tools are not universally applicable and come with substantial caveats. GenAI integration into ecological workflows has implications for the kinds of questions we ask, the methods we use, and ultimately what ecology looks like as a field and as a profession. Overreliance on GenAI risks narrowing intellectual exploration, reinforcing existing biases, and displacing the creative, theory‐driven, and observational foundations that have long defined ecological science. In this sense, the challenge is cultural: how to adopt new tools without eroding the practices that foster insight, skepticism, and discovery. In our view, the next generation of scientists will be defined by their command of theory and their ability to think creatively about complex problems, which are capacities that remain, at least for now, resistant to automation.

More broadly, the rapid push toward optimization in science invites a deeper question: what do we value in the process of doing science? In the face of global climate change, species declines, anthropogenic pressures, and resource management concerns, it is perhaps more urgent than ever before for ecologists to respond to management needs quickly and rigorously (Meinke et al. 2009; Turner et al. 2020). Yet we caution against efficiency for efficiency's sake and argue that just because we could automate portions of the scientific process does not mean that we ought to. Many ecologists are drawn to the field by a desire to engage deeply with natural systems, to ask meaningful questions, and to contribute knowledge that matters. If too much of this process is outsourced to GenAI tools, the sense of purpose and satisfaction derived from scientific work may be diminished. We advocate for careful reflection on how GenAI can be integrated without compromising the aspects of our work that provide fulfillment, enjoyment, and purpose.

It is also likely that GenAI adoption will vary widely across the field. Some researchers may embrace these tools, while others may opt out due to access limitations, personal and ethical considerations, or simply lack of relevance to their research interests (Docter‐Loeb 2026; Shata 2025; Yang et al. 2026). Maintaining a diverse and inclusive scientific community will require recognizing and explicitly supporting this range of engagement. Funding structures, hiring practices, and institutional policies should avoid privileging speed or productivity alone, and instead broadly and holistically value rigor, creativity, and multiple ways of doing science.

Ultimately, ecologists want to think about ecology. GenAI offers tools to free up cognitive burden, reduce busy work, and create space for us to focus on questions of fundamental and applied significance. Yet it may also reduce the need or inclination to think creatively and deeply. Navigating this tension will require deliberate choices about how these tools are integrated into our work. As a field, now is the time to reflect on and advocate for the kinds of engagement we value and ensure that GenAI supports, rather than displaces, the kinds of thinking that underpin ecological insight and discovery.

## Author Contributions

**Nina C. Ferrari:** conceptualization (equal), investigation (lead), visualization (equal), writing – original draft (lead), writing – review and editing (equal). **Emily E. Conklin:** conceptualization (equal), investigation (lead), visualization (equal), writing – original draft (lead), writing – review and editing (equal). **Amelia A. Fitch:** conceptualization (equal), investigation (supporting), writing – original draft (supporting), writing – review and editing (equal). **Dustin G. Gannon:** conceptualization (equal), investigation (lead), writing – original draft (supporting), writing – review and editing (equal). **Rossana Macedo:** conceptualization (equal), investigation (supporting), writing – original draft (supporting), writing – review and editing (equal). **Aidan Place:** conceptualization (equal), investigation (supporting), writing – original draft (supporting), writing – review and editing (equal). **Hannah R. Sachs:** conceptualization (equal), investigation (supporting), writing – original draft (supporting), writing – review and editing (equal). **Eugene Seo:** conceptualization (equal), investigation (supporting), writing – original draft (supporting), writing – review and editing (equal). **Matthew J. Weldy:** conceptualization (equal), investigation (supporting), writing – original draft (supporting), writing – review and editing (equal). **Matthew G. Betts:** conceptualization (equal), funding acquisition (equal), investigation (equal), supervision (equal), writing – original draft (supporting), writing – review and editing (equal).

## Funding

This work was funded by the National Science Foundation Long‐term Ecological Research (LTER) grant DEB‐2025755 and National Science Foundation grant number 2234662.

## Conflicts of Interest

The authors declare no conflicts of interest.

## Data Availability

The authors have nothing to report.
